# Cancellation of the vestibulo-ocular reflex during smooth pursuit in patients with maculopathy

**DOI:** 10.3389/fneur.2025.1632527

**Published:** 2026-01-14

**Authors:** Natela M. Shanidze, Anca Velisar

**Affiliations:** The Smith-Kettlewell Eye Research Institute, San Francisco, CA, United States

**Keywords:** macular degeneration, central field loss, vestibuloocular reflex, VOR cancellation, smooth pursuit, eye-head coordination

## Abstract

**Introduction:**

Macular degeneration is associated with loss of central vision, including the fovea. Central visual field loss is associated with impaired smooth pursuit eye movements, and the associated scotoma can lead to target disappearance which may or may not be transient. While smooth pursuit in and of itself does not require a foveal position signal, suppression of the vestibuloocular reflex (VOR) needed during combined eye and head pursuit relies on the fovea for appropriate calibration. Prior work has shown that target extinction during, or even at the beginning of the trial does not affect the dynamics of the eye or head responses necessary for pursuit. These findings suggest that the potential disappearance of the target into the scotoma during pursuit can be overcome, particularly when both eye and head movements are used. The objective of this study was to test the hypothesis that head-unrestrained smooth pursuit deficits in CFL are at least partially related to individuals’ inability to cancel their VOR.

**Methods:**

We performed a secondary analysis of a previously published data set of head restrained and unrestrained pursuit of a 10 °/s target in 7 individuals with maculopathy (56-89 years) and 7 age-matched controls (61-78 years). We used a two-parameter linear regression model to quantify the eye-only pursuit (K_fix_) and VOR (K_v_) contributions to determine whether participants were able to effectively cancel the VOR to improve pursuit gain.

**Results:**

We observed reduced pursuit velocities in the head-unrestrained versus restrained smooth pursuit condition (K_fix_ < 1), particularly in the control group (CFL: K_fix_ = 0.90 ± 0.16; Control: K_fix_ = 0.74 ± 0.08). For VOR cancellation, K_v_ estimates did not deviate from -1 for either of our groups (CFL: K_v_ = -0.92 ± 0.09; Control: K_v_ = -0.98 ± 0.20), suggesting complete cancellation of the VOR during head-unrestrained pursuit. For the self-generated VOR experiment, participants in both groups had similar, near-one VOR gains (CFL: Gain_VOR_ = -1.00 ± 0.05; Control: Gain_VOR_ = -1.03 ± 0.02).

**Discussion:**

We confirmed that VOR responses to actively generated head movements were similarly intact in both groups. Our analyses of the pursuit data indicate that, regardless of visual function older adults attenuate eye-driven pursuit when the head is unrestrained (as compared to the restrained condition), and those with CFL fail to exploit residual VOR cancellation, helping to explain their persistent gaze-tracking deficits.

## 1 Introduction

Age-related macular degeneration (AMD) occurs in over 7 million individuals in the United States alone (1, 2), and is the most common cause of central visual field loss (CFL). Loss of vision in general, and foveal vision in particular, has significant implications for patients’ quality of life (3, 4), and presents risks such as falling (5–7), which can be fatal in older adults (8). While there has been substantial research on the consequences of losing central fixation for visual function (4, 9, 10) and oculomotor behaviors during reading (11), there has been little focus on the coordination of eye and head movements in individuals without a fovea.

In individuals with CFL, the quality and control of eye movements are impaired (12, 13). The reasons for this deficiency are complex. First, CFL is typically associated with the loss of the fovea as the oculomotor reference. Although the fixational preferred retinal locus (PRL) has been shown to develop into an oculomotor reference for saccades (14), catch-up saccades in smooth pursuit (15), and possibly vestibuloocular reflex (VOR) at near viewing (16), this process is slow, and individuals do not always rely on a single eccentric PRL. Indeed, individuals with CFL can switch between different loci on the retina during smooth pursuit (17). Furthermore, how an eccentric retinal reference would be coordinated with head movements is not well understood.

Beyond oculomotor considerations, visual information can also be impaired during eye movements. Smoothly-moving objects can disappear and reappear in the patient’s scotoma – the area where the visual field is no longer functional. However, unlike an external occluder, patients are typically not aware of their scotoma (18). Indeed, in a recent study Rubenstein and colleagues (2025) (19) showed that both target eccentricity and intermittent target occlusion affected motion extrapolation in macular degeneration. This work is consistent with our own studies showing that smooth pursuit is impaired (17), gain varies with target-scotoma trajectory (17), catch-up saccades are often misdirected (20), and dynamic visual acuity is impaired during pursuit (21) in individuals with CFL.

Prior work in healthy sighted participants has shown that smooth pursuit is driven by both visual signals from the retina and internal drive mechanisms, which are extraretinal in nature (22) and particularly effective when there is an expectation of target reappearance (23). In head free pursuit, this extraretinal component is the main driver of head velocity (24). Indeed, head movements can enhance gaze pursuit velocity in cases of target disappearance, assuming a partial cancellation of the VOR (25).

Based on work by Ackerley and Barnes (25), we thus hypothesized that some pursuit deficits in CFL may be alleviated if individuals used both eye and head movements to pursue smoothly moving targets. However, when we examined pursuit behavior in individuals with and without CFL who used eye-only or eye and head movements to pursue targets, we did not observe an improvement in pursuit gain in the head-unrestrained condition (26). In that study, participants with CFL had lower pursuit gains than controls, despite having similar head movement amplitudes. This outcome led us to wonder if the benefits of head-free pursuit were countermanded by deficits of eye-head coordination, particularly VOR suppression (or cancellation) in our CFL cohort.

During large gaze shifts, or eye and head tracking of a moving object, the VOR can be counterproductive, taking the eyes away from the target of interest. Numerous studies suggest that VOR cancellation has both visual and nonvisual components, with the visually driven contribution improving the precision of the response at smooth pursuit latencies (27). Indeed, there is a strong association between pursuit and VOR cancellation in humans (28), consistent with shared pathways for smooth pursuit and VOR cancellation (29). In this study, we set out to assess whether head-based pursuit and VOR cancellation can be a useful adaptation in CFL participants where target disappearance and other disease-related factors could lead to a less effective visual (retinal) pursuit signal than in healthy-sighted controls (26). Using the approach developed by Lefèvre et al. (30), and expanded to pursuit (25), we modeled the head-unrestrained pursuit as a combination of uncompensated head and smooth pursuit eye movements to determine their relative contributions.

We modeled gaze velocity during head free pursuit ( ${\dot{EIS}}_{hFree}$ , eye-in-space) as a combination of eye velocity generated during head-restrained pursuit ( ${\dot{EIS}}_{hFix}$ ) and the sum of the head movements ( $\dot{HIS}$ ) and the compensatory eye-in-head response ( $K_{v}*\dot{HIS}$ , where $K_{v}$ is negative, representing the counterrotation of the eye opposite the head, Equation 1).


(1) $${\dot{EIS}}_{hFree}=K_{fix}*{\dot{EIS}}_{hFix}+\left(1+K_{v}\right)*\dot{HIS}$$

In this formulation, the $K_{fix}$ coefficient represents the attenuation in the eye velocity command between the head-unrestrained and head-restrained conditions, as the two would be equivalent prior to head movement onset if $K_{fix}=1$ . The $K_{v}$ coefficient is related to the contribution of the head movement to overall gaze velocity and accounts for the magnitude of the vestibuloocular reflex. If the VOR is completely compensatory, $K_{v}=-1$ , the head movement is entirely canceled by the VOR response and the entire $\dot{HIS}$ term would go to zero (there would be no head contribution to pursuit gaze velocity). Any cancellation, or attenuation of the VOR would therefore be accounted for by an increase in the $K_{v}$ coefficient to be $0\geq K_{v}>-1$ .

We hypothesized that in our participants we would find $K_{fix}≅1$ , as had been shown previously for participants pursuing targets that were either always visible or briefly extinguished during the trial (24, 25). In controls, we also expected to observe $K_{v}≅-1$ , akin to the trials where the target was always visible in (24, 25) and pursuit gains were near-optimal due to an intact retinal slip command. In participants with CFL, however, we hypothesized that we would not observe the 15% reduction in VOR, corresponding to a $K_{v}=-0.85$ reported for the extinction trials in (24, 25). Instead, despite lower pursuit gains and intermittent target disappearance in the scotoma, we expected to find $K_{v}≅-1$ , suggesting that the head movements did not help improve pursuit performance in CFL due to a lack of VOR cancellation.

## 2 Methods

All research was performed in accordance with the Declaration of Helsinki and was approved by the Institutional Review Board of the Smith-Kettlewell Eye Research Institute. All participants gave their written informed consent prior to participation.

All data processing and analyses were done using custom software written in MATLAB (MathWorks, Natick, United States).

### 2.1 Smooth pursuit

#### 2.1.1 Participants and task

Smooth pursuit data were collected as part of a previous study of head-restrained and unrestrained smooth pursuit in individuals with macular degeneration (26). Eight participants with maculopathy (age: 56–89 years, 4F) and 7 older controls (age: 61–78, 7F) participated in that study (for full demographic information, see Table 1 in (26), or Supplementary Table S&V1). Briefly, participants were seated 1 meter from a large monitor and were asked to pursue a one-degree (visual angle) target moving in one of two horizontal or two vertical directions. The target moved in a modified step-ramp, jumping 6° in one of four possible directions from the central fixation and smoothly moving in the opposite direction through the center of the screen at 10°/s. Target directions were randomized across trials. Participants performed 15 trials per each trial direction, for a total of 60 trials. While seated, participants were either placed in a chin and forehead rest (head-restrained condition) or were asked to sit upright and pursue the target as they would naturally (26).

#### 2.1.2 Data recording and processing

Briefly, eye movements were recorded binocularly using Pupil Core binocular infrared eye tracking goggles (Pupil Labs, Munich, Germany) sampling at 200 Hz for each eye. Head movements were recorded using a nine-axis inertial measurement unit (IMU; LP Research, Tokyo, Japan) attached to the scene camera of the eye tracking goggles, sampling at 400 Hz (100 Hz for participants P6 and P8). The IMU was aligned with the eye tracker scene camera such that their reference frames had similar orientation. The IMU recorded the head motion and the orientation of the scene camera relative to the world (global reference). The eye tacker estimated the gaze vectors of the two eyes, represented in the scene camera 3D reference frame. The gaze vectors estimates were corrected using a homography calculated for each participant using data from a validation task in which the participants fixated markers on a 13-point grid in sequential order. The gaze vectors were then transformed to the global reference frame using the IMU sensor orientation data. The head and gaze data were resampled to 1,000 Hz. Saccades were removed from the gaze data using an eye acceleration-based detection algorithm and confirmed by an observer. The velocity traces of trials with excessive continuous data loss due to artifacts or pupil occlusions were rejected for future analysis. Additionally, the gaze data was interpolated to remove short data gaps due to blinks or the elimination of saccades. The head and gaze data were filtered with a two-pole Butterworth noncausal filter (cutoff 25 Hz). For detailed methods, see (26).

#### 2.1.3 Smooth pursuit and VOR cancellation modeling

Data from 7 participants with CFL (mean age: 75) and 7 controls (mean age: 72) was used for further analysis [participant P6 in Table 1 of (26) was excluded due to frequent data artifacts at pursuit initiation]. Gaze data from the eye with the most valid samples across trials was chosen for each participant.

For each head restraint condition, the gaze velocity and head angular velocity was averaged over all trials in a given target direction. Prior to this step, the trials in head-unrestrained condition in which the maximum head angular velocity did not exceed 2°/s were removed from the analysis. This threshold based on the maximum head velocity across all pre-trial fixations, as anything under that was below optimal velocity and acceleration thresholds of the semicircular canals (31) and deemed to be natural head drift and not gaze shift related. This step allowed us to estimate both the eye movement (K_fix_) and uncompensated head contribution to pursuit (K_v_) together (see Equation 2). Each average trace was then filtered with a four-pole Butterworth Zero-phase filter. A baseline was calculated for the gaze and head movement traces during the fixation period and subtracted from the traces. Eye-in-head time series during the head-unrestrained condition was calculated by subtracting the head movement data from the gaze estimates.

**Figure 1:**
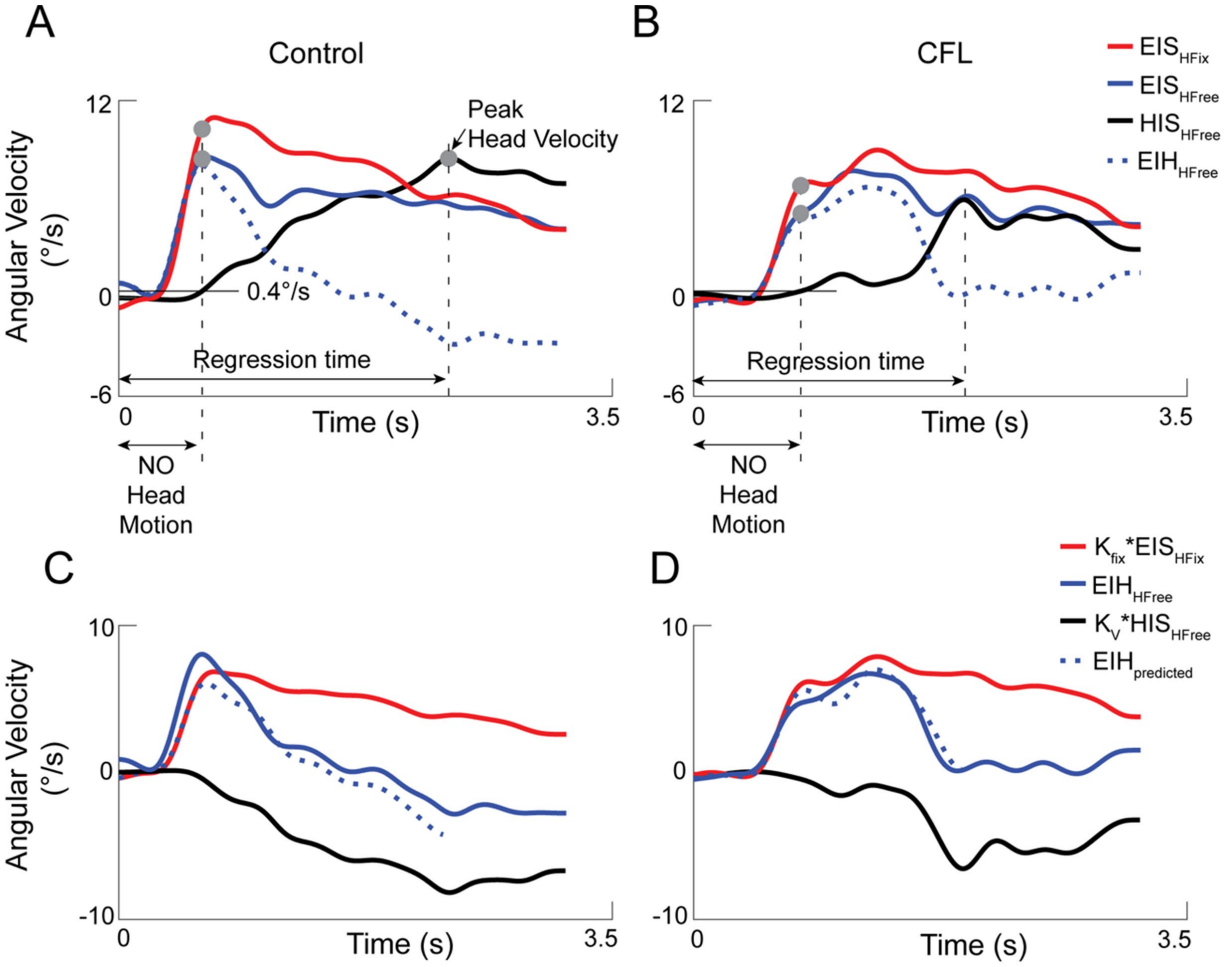
Individual set of angular velocity time series of gaze (EIS, blue), eye in head (EIH, dotted blue) and head (HIS, black) data for the head-unrestrained condition and gaze (red) for the head-restrained condition for control **(A)** and CFL **(B)** participants. Traces are averaged across all rightward trials for a single participant. The regression time was chosen for each participant from the start of target motion to the time of peak head velocity. The “No Head Motion” period at the beginning of the trial is defined as the time when the head velocity is less than 0.4°/s threshold. The gaze velocities at the end of the “no head motion” period are represented by gray dots placed on respective gaze velocity signals. **(C,D)** The non-compensatory (K_fix_*EIS_hFix_, red) and compensatory components (K_v_*HIS_hFree_, black) of the eye movement model in smooth pursuit, in addition to the measured EIH_hFree_ (blue) and EIH_predicted_ (dotted blue) velocities for control **(C)** and CFL **(D)**. All graphs represent data from horizontal smooth pursuit trials (0°).

Using Equation 1, which solves for $\dot{EIS}$ , we used the relationship $\dot{EIS}=\dot{EIH}+\dot{HIS}$ to solve for eye in head ( $\dot{EIH}$ ). We then constructed a multiple regression model where eye-in-head velocity ( $\dot{EIH}$ ) during head-free pursuit is equal to the weighted sum of eye-in-head ( $\dot{EIH}$ ) velocity during head-fixed pursuit (also gaze, due to lack of head movement) and delayed head velocity during head-free pursuit (Equation 2; Figures 1C,D).


(2) $${\dot{EIH}}_{hFree}=K_{fix}{\dot{EIH}}_{hfix}+K_{v}\dot{HIS}$$

The regression time was chosen for each participant over each direction as the time from start target motion to peak head angular velocity during the head-restrained condition. K_fix_, K_v_ and the delay were calculated as in (25) for each participant (Figures 1A,B).

Head movement onset was estimated by finding angular head velocity that was greater than all head velocities measured in the head-restrained condition. Using this method, head movement threshold was set at 0.4°/s as the start of head motion in the head-unrestrained condition (Figure 1 A&B). Gaze velocities at the start of head motion were compared across head restraint conditions.

We calculated the time of maximum gaze acceleration for both head conditions and extracted corresponding gaze velocities at these times. The lower of the two velocities (between the two conditions) was chosen as the gaze velocity threshold. The difference between the time of peak gaze acceleration and the time where gaze velocity crossed the threshold was considered the latency between pursuit velocities in the two head restraint conditions.

### 2.2 Vestibuloocular reflex

#### 2.2.1 Participants and task

In a separate set of experiments, 6 participants with maculopathy (age: 61–82 years, 4F) and 4 age-matched controls (60–81, 4F) were recruited to perform an active vestibuloocular reflex (VOR) task (see Table 1 for demographics). The self-generated head movement task was chosen to be more closely analogous to VOR during pursuit, as active gaze stabilization during active head movement differs from conventional VOR (32–34). VP1, VP2, and VP3 were participants P2, P3, and P8 from the original study, respectively (see Table 1 in (26) or Supplementary Table S&V1). In the intervening years between experiments, VP1 had progressed to have binocular scotomas. While disease progression was less profound in the other two, VP2 did switch to using a different PRL.

**Table 1:** Vestibuloocular reflex experiment participant summary.

ID	Age	Sex	Diagnosis	LogMAR visual acuity, [corrected]	Mars contrast sensitivity	Dominant eye PRL eccentricity (°)
P1	82	F	AMD	0.68	1.5	1.80°
P2	80	F	AMD	0.04	1.4	0.95°
P3	61	M	Stargardt’s	0.94	0.9	5.55°
P4	79	M	AMD (monocular)	0.10	1.7	0°
P5	64	F	Retinal Detachment (monocular)	0.20	1.7	0°
P6	82	F	AMD (monocular)	0.24	1.4	0°
C1	75	F	Control	0.30 [0.04]	1.8	0°
C2	60	F	Control	0.20 [0.02]	1.8	0°
C3	81	F	Control	0.12	1.8	0°
C4	74	F	Control	0.10	1.6	0°

*Visual acuity and contrast sensitivity reported for binocular viewing.

#### 2.2.2 Data recording and processing

Participants wore the EyeSeeCamSci 2 binocular eye-tracking goggles (EyeSeeTec GmBH, Berlin, Germany, sampling rate: 250 Hz for each eye) and were asked to make horizontal sinusoidal head rotations at 0.5 Hz, to the sound of a metronome, while fixating an LED target at 2 meters. Participants were instructed to rotate their head “clavicle to clavicle,” after which they watched a demonstration of the behavior and were then asked to practice it several times before starting the experiment.

The EyeSeeCamSci 2 eye tracker has an integrated IMU, such that the device simultaneously records eye-in-head position and velocity, head-in-space angular velocity, linear acceleration and orientation. Offline, saccades, blinks and other discontinuities were removed manually from the eye signals and the data gaps were filled by interpolation. Head and eye data was filtered with a two-pole Butterworth non-causal filter (cutoff 3 Hz). A linear regression analysis between the eye and head angular velocity was performed to determine the VOR gain for each eye. Here, we report the average VOR gain between the two eyes.

### 2.3 Statistics

Normality was tested using the Shapiro–Wilk test. Alpha was set at 0.05. For multiple comparisons, *p*-values were adjusted using the Šídák test for multiple comparisons. All secondary statistical analyses were performed using Prism 10 software (GraphPad Software LLC.).

## 3 Results

### 3.1 Qualitative observations

**Figure 2:**
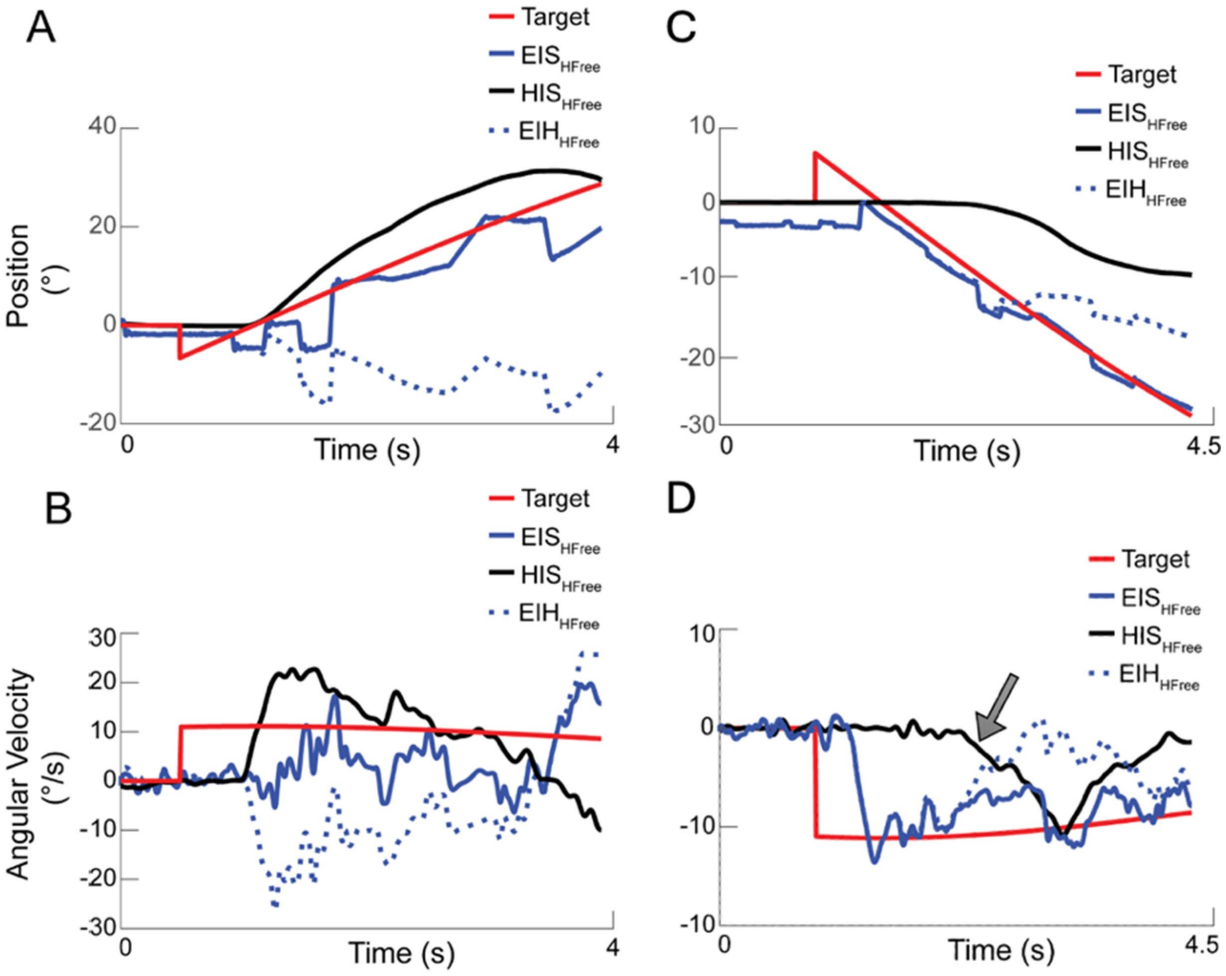
Example individual trials from participants P2 **(A,B)** and P3 **(C,D)** from (26). Both position **(A,C)** and velocities **(B,D)** of the head (black trace), eyes-in-head (dotted blue trace), gaze (blue trace), and target (red trace) are shown.

Figure 2 shows two example head-unrestrained trials from participants P2 (Figures 2A,B) and P3 (Figures 2C,D) from the original study (Supplementary Table S&V1).

P2 typically did not initiate pursuit with the eyes prior to moving the head. When the head did begin to move, however, she was not able to sufficiently cancel the VOR for the duration of the trial, with eye-in-space (gaze) velocity being less than target velocity for most of the trial (Figure 2B). At the end of the trial, the head moved in the opposite direction of the target with a clear compensatory eye movement moving the eye in the opposite direction of the head (but now with the target). The position of the eyes in the head remained relatively constant.

Participant P3 initiated pursuit with the eyes only and moved the head later in the trial (possibly to increase pursuit range). At the initial acceleration of the head (Figure 2D, gray arrow), the eyes moved in a compensatory direction, and the overall gaze velocity was reduced. However, as the trial went on, the participant was better able to coordinate her eye and head movements, and gaze velocity returned to be closer to target velocity.

### 3.2 Modeling the data: pursuit and VOR coefficients

Figure 1 shows representative average traces of eye, head, and gaze velocities across all trials for the rightward (0°) direction for the head unrestrained (blue and black lines) and head-restrained (red) conditions. Key features to note include the initial pursuit by the eyes only (marked as “NO head motion”), higher gaze velocity in the head-restrained than unrestrained condition for the control participant and the eventual reduction of the eye-in-head velocity with the increase in head velocity in the head-unrestrained condition.

We characterized the eye motion during head-unrestrained pursuit as a combination of a scaled (K_fix_) smooth pursuit eye command for the head-restrained condition and delayed compensatory response to head motion (K_v_, see Methods, Section 2.3, Figures 1C,D). The eye and head time series used in each model for each participant in each direction lasted from target onset to the timepoint of peak head velocity.

**Figure 3:**
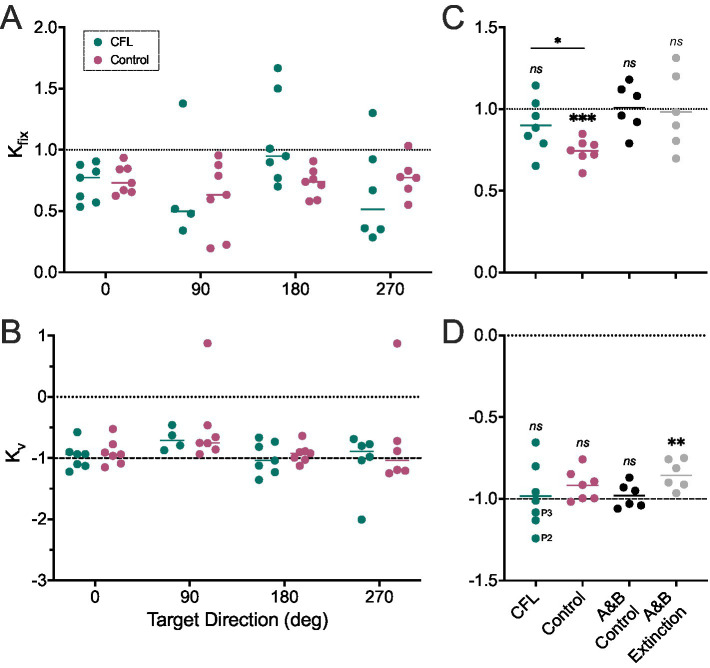
K_fix_ **(A,C)** and K_v_ **(B,D)** estimates for participants with CFL (green) and controls (magenta). **(A,B)** Each dot represents a coefficient estimate for one direction, for one participant. **(B,D)** Coefficients are averaged across the horizontal (0° and 180°) directions and shown next to analogous coefficients from (25) for trials with (gray) and without (black) target extinction. Statistical comparisons are shown for one sample *t*-test comparing against 1 (K_fix_) and −1 (K_v_) as well as comparing between our participant groups (K_fix_). NS: Not significant; *p < 0.05; **p < 0.01; ***p < 0.001. Dashed lines: expected coefficient value.

Because target direction can significantly affect pursuit gain in individuals with CFL (17), we provide pursuit and VOR coefficient estimates for all directions individually in Figures 3A,B. Although we saw a trend for lower K_fix_ values for the vertical directions, there was no significant effect of direction [two-way mixed effects model with Geisser–Greenhouse sphericity correction, K_fix_: *F* (1.45, 20.82) = 1.95, *p* = 0.17; K_v_: *F* (1.25, 12.89) = 1.96, *p* = 0.19]. Further, there was no effect of participant group [K_fix_: *F* (1, 32) = 0.66, *p* = 0.42, 95% CI: (−0.094, 0.222); K_v_: *F* (1, 12) = 1.10, *p* = 0.32, 95% CI: (−0.473, 0.166)]. For additional statistical parameters, see Supplementary Table S1.

While the effect of direction was not significant, the horizontal and vertical trials were not equivalent, with shorter trajectories and trial durations for the vertical directions. Therefore, we focused on the horizontal directions, for a more direct comparison to prior work (24, 25). Having averaged the leftward and rightward conditions, we observed K_fix_ < 1 (i.e., a reduction in the smooth pursuit velocity for head-free versus head-restrained pursuit; CFL: K_fix_ = 0.90 ± 0.16; Control: K_fix_ = 0.74 ± 0.08; Figure 3C) for both groups, unlike in (25) (Control: K_fix_ = 1.01 ± 0.14, Extinction: K_fix_ = 0.98 ± 0.23, black and gray dots respectively).

To determine whether this reduction was indeed significant on a group level, we performed a one-sample *t*-test (Table 2) which indicated that our older adult control group had K_fix_ estimates significantly below 1 (suggesting different eye pursuit velocities between the head-free and head-fixed conditions, Row 1, K_fix_ columns, Table 2). We did not find a significant difference for the CFL group (Row 2, K_fix_ columns, Table 2) or the K_fix_ estimates from (25) (Rows 3 and 4, K_fix_ columns, Table 2). We did find a significant difference in K_fix_ estimates between our two groups (two-tailed *t*-test, Row 5, Table 2).

**Table 2:** Statistical parameters and outcomes for K_fix_ and K_v_.

Participant Group	K_fix_ = 1				K_v_ = −1			
	Mean ± SD	*t*-statistic, *p*-value	95% CI	Cohen’s d, N	Mean ± SD	*t*-statistic, *p*-value	95% CI	Cohen’s d, N
Control	0.744 ± 0.075	8.93, ***<0.001***	[−0.325, −0.187]	3.44, 7	−0.918 ± 0.094	2.31, *0.06*	[−0.005, 0.169]	0.87, 7
CFL	0.907 ± 0.155	1.63, *0.15*	[−0.236, 0.050]	0.60, 7	−0.983 ± 0.200	0.23, *0.83*	[−0.168, 0.203]	0.09, 7
A&B 2011 Control	1.008 ± 0.145	0.14, *0.89*	[−0.144, 0.160]	0.06, 6	−0.980 ± 0.075	0.65, *0.54*	[−0.059, 0.099]	0.27, 6
A&B 2011 Extinction	0.983 ± 0.235	0.18, *0.87*	[−0.264, 0.229]	0.07, 6	−0.850 ± 0.089	4.14, ***0.009***	[0.057, 0.244]	1.69, 6
Control vs. CFL	0.744 ± 0.075 vs. 0.907 ± 0.155	2.30, ***0.04***	[−0.304, −0.008]	1.34, 7 vs. 7	−0.918 ± 0.094 vs. -0.983 ± 0.200	0.78, *0.45*	[−0.117, 0.247]	0.42, 7 vs. 7

* Rows 1–4, one-sample *t*-tests comparing our data (Rows 1, 2) and prior results from Ackerley and Barnes (25) (Rows 3, 4) to ideal (K_fix_ = 1 and K_v_ = −1). Row 5, two-tailed, unpaired t-test for group comparisons (Control vs. CFL) of K_fix_ and K_v_. SD: standard deviation, CI: confidence interval, N: sample size. Bolded values indicate significant statistical outcomes.

Examining K_v_ estimates, on the other hand (Figure 3D), we did not observe a deviation from K_v_ = −1 (i.e., VOR gain = 1) for either of our groups (one-sample *t*-test, Table 2, K_v_ columns, Rows 1 and 2), similar to the control (non-extinction) values in (25) (Table 2, K_v_ columns, Row 3) and unlike the extinction condition in (25), where the authors reported K_v_ = −0.85 ± 0.10, corresponding to a 15% cancellation of the VOR during pursuit (Table 2, K_v_ columns, Row 4). We did not find a significant difference in the K_v_ coefficients between our two participant groups (Row 5, Table 2). Additionally, there was no difference between our control participants and the control condition in Ackerley and Barnes (25), one-way ANOVA with a Holm-Šídák’s multiple comparisons test, *p* = 0.73. Participants with individual trials shown in Figure 2 are marked in Figure 3D for reference.

### 3.3 Gaze velocity

**Figure 4:**
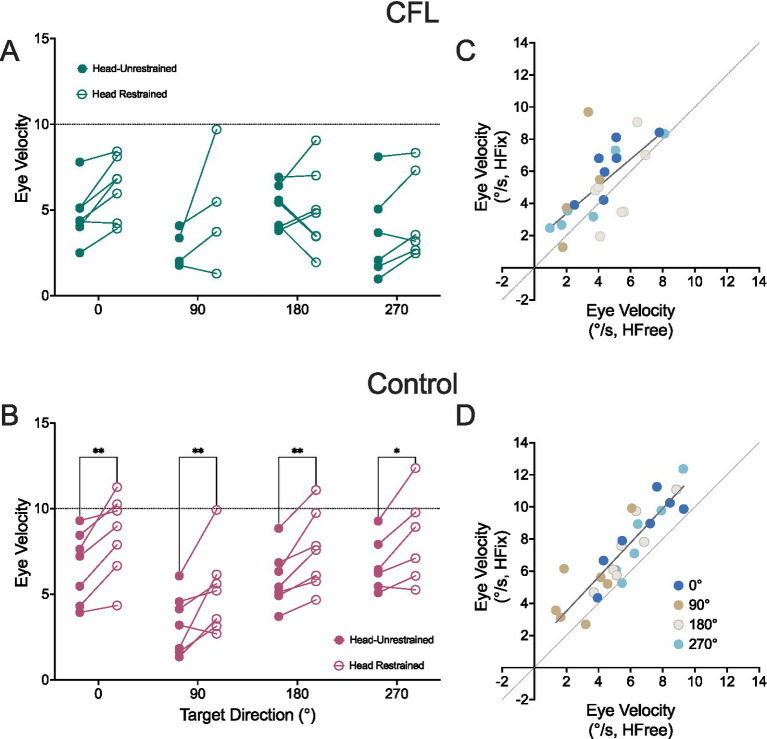
Eye-in-space velocities calculated immediately before head movement onset threshold for the head-restrained and unrestrained conditions for participants with CFL **(A,C)** and controls **(B,D)**. Gray dashed line: line of identity, slate solid line: regression line fit to data combined across all target directions (denoted as blue: 0°– rightward, tan: 90°– downward, gray-pink: 180°– leftward, and teal: 270°– upward).

Estimates of K_fix_ < 1 suggest that the *eye* velocity response in the two head restraint conditions was different. To test this interpretation, we calculated eye-in-space velocity immediately prior to head movement onset for each trial, where pursuit would already be initiated, but no head movement contribution can yet occur (see Methods, Section 2.3). These values were then averaged for each target direction, for each participant. Figure 4 shows eye velocities in the head-free condition compared with eye velocities in the head-restrained condition for each CFL (Figures 4A,C) and control (Figures 4B,D) participant.

To determine whether eye velocities prior to head movement were indeed different from eye velocities in the head-restrained condition at that same timepoint, we performed a two-way repeated measures ANOVA and found a significant effect of head condition for both CFL [*F* (1, 20) = 10.18, *p* = 0.005, 95% CI: (−1.900, −0.398), η_p_^2^ = 0.34] and control [*F* (1, 23) = 49.17, *p* < 0.0001, 95% CI: (−2.248, −1.224), η_p_^2^ = 0.68] groups. We subsequently performed a Šídák test for multiple comparisons of head-restraint conditions at each target direction. We found no individual target direction effects for the CFL group (*p* > 0.07). In the control group, there was a significant difference between head restraint conditions for each target direction (Šídák multiple comparisons test, adjusted *p*-values: 0°: *p* = 0.004, 90°: *p* = 0.002, 180°: *p* = 0.01, 270°: *p* = 0.03).

To ascertain if this head-restraint-based change in eye velocity was velocity dependent, we performed a simple linear regression for each direction and each group. In the CFL group, the slopes all ranged between 0.86 (0°, rightward) and 2.23 (90°, downward) and were not significantly different upon comparison [*F* (4, 33) = 0.55, *p* = 0.70]. We found a similar trend in the control group, where slopes ranged between 1.01 (0°, rightward) and 1.60 (270°, upward) and were not significantly different from each other [*F* (4, 34) = 0.6376, *p* = 0.64]. Thus, we calculated a single slope for each group, both of which were near-one (CFL: slope = 0.85, s.e. = 0.20; Control: slope = 1.07, s.e. = 0.11, Figures 4C,D respectively). There was no significant slope difference between groups [*F* (1,47) = 0.98, *p* = 0.33]. See Supplementary Table S2 for detailed fit statistics.

One possible explanation of the difference in eye velocities between conditions is a difference in pursuit latencies – a longer pursuit latency in the head-unrestrained condition could lead to lower eye velocities. To test this possibility, we calculated pursuit latency offset between the two conditions for both groups. Median pursuit latencies were exceedingly close to 0 for both groups (CFL: −0.0095 s, Control: 0.004 s, One-Sample Wilcoxon test, *p* > 0.25, W_Control_ = 33, W_CFL_ = −78).

### 3.4 Vestibuloocular reflex to active head movements

**Figure 5:**
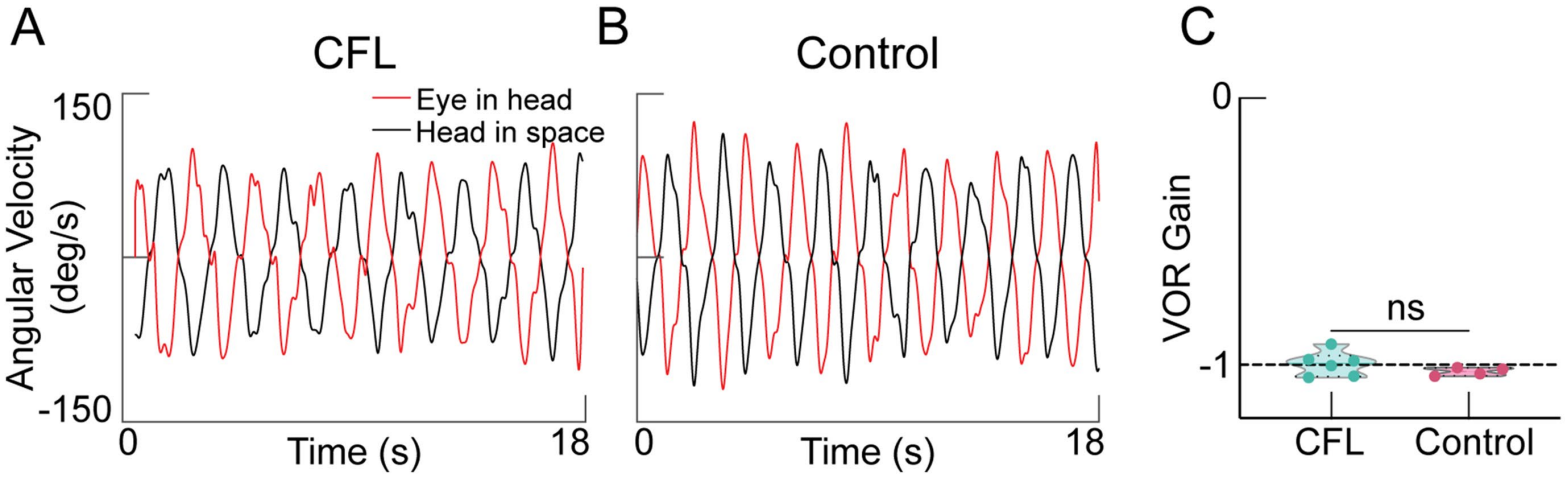
Active VOR responses to self-generated head movements. Eye and head velocity data for participants P5 [maculopathy, **(A)**] and C2 [control, **(B)**] making head rotations at 0.5 Hz, while viewing an LED target at 2 meters. **(C)** VOR gains across the maculopathy (turquoise) and control (red) groups are both nearly at the ideal of −1 (dashed line).

Given the limited published data on VOR in CFL, we performed an additional experiment where we asked participants to rotate their heads periodically while viewing an LED dot stimulus at a viewing distance of 2 meters. Figures 5A,B shows representative velocity traces for the eye-in-head (red) and head-in-space (black) from a participant with CFL (VP5, Table 1) and a control (VC2, Table 1) participant. Both show similar eye and head movement profiles with a near-equal and opposite rotation of the head and eyes in orbit. Indeed, we observed similar (near-one) gains for both groups [Figure 5C, CFL: Gain_VOR_ = −1.00 ± 0.05; Control: Gain_VOR_ = −1.03 ± 0.02; *t* = 1.12, *p* = 0.27, 95% CI = (−0.085, 0.027), Cohen’s d = 0.754].

## 4 Discussion

In this study, we investigated the hypothesis that individuals with central visual field loss do not effectively use head movements to enhance their horizontal or vertical smooth pursuit performance due to a lack of VOR cancellation. While there was substantial variability in eye-head coordination behavior in the CFL group, overall, we did not observe lower VOR gains consistent with VOR cancellation needed to enhance deficient pursuit velocities. Indeed, this lack of VOR cancellation may have contributed to further decreases in gaze velocities we reported in (26). Interestingly, the control group had lower pursuit eye velocities prior to head movement initiation when the head was unrestrained, than when the head was restrained, potentially causing an overall decrease in pursuit velocity observed during head-unrestrained pursuit (26).

### 4.1 Comparison to prior work

In our study (26) of head-unrestrained smooth pursuit in individuals with CFL, we found group differences between eye, but not head, velocities during pursuit, which were also evident in the overall gaze gain during pursuit. These differences persisted in both head-restrained and head-unrestrained conditions (26). Moreover, we found that in the head-unrestrained version of the task, eye velocities in the CFL group were not only significantly lower than in the head-restrained condition but were sometimes negative [Figure 3A in (26), black circles], suggesting that the eyes were moving opposite the target. We did not observe these oppositely directed movements for the head velocity, however. Thus, the eyes were moving not only opposite the target, but also the head – a behavior suggestive of VOR. Indeed, when we examined this behavior on an individual trial basis, we observed that head velocities were sometimes accompanied by compensatory eye-in-head movements that appeared to be equal and opposite the head. In both the control and CFL groups, we found that overall gaze velocities (and therefore pursuit gains) were lower in the head-unrestrained, than restrained condition, with pursuit gains in CFL participants being lower than in controls, for both conditions (Figure 1 of the original study).

We hypothesized that the lower pursuit gains in the CFL group in both head-restraint conditions were likely due to a combination of oculomotor deficits and target disappearance in the scotoma (19). Previously, Ackerley and Barnes (24, 25) showed that participants could extract target motion information from a brief presentation to appropriately scale gaze velocity (25). In the extinction trials, the authors saw a contribution of ~15% uncompensated-for head movements to pursuit of temporarily extinguished targets for an overall improvement of pursuit gaze velocities (25). From our own and others’ work, we know that motion perception is generally not impaired in CFL (35, 36).

Further, Ackerley and Barnes found that target extinction duration or whether it was blanked at the beginning of or during the trial did not have a significant effect on either K_v_ or K_fix_ estimates (Equation 1) (24, 25). This outcome would suggest that the variability we see in our CFL participants may not be directly attributable to their inability to keep the target visible at various stages of the trial during head-unrestrained pursuit. In other words, given the highly repeatable nature of our trials and constant target velocity during the ramp, participants with CFL should be able to sustain gaze velocity using efferent/memory information. Taken together, these outcomes suggest that head pursuit in conjunction with VOR suppression can provide an effective adaptation to overcome pursuit deficits due to intermittent target disappearance in CFL.

However, while in the prior study (25), when targets were extinguished during a part of the trial, VOR suppression was estimated to be ~15%; CFL participants in our study had on average near-one VOR gains (K_v_ = −0.98), suggesting that the head movements were nearly-perfectly compensated for by the eyes in orbit. This strategy made head movements an ineffective contribution to smooth pursuit in CFL. Interestingly, in our control participants we did observe K_v_ values that were slightly greater than those estimated by (25) for their control (no extinction) trials. However, overall, this difference was not significant.

An interesting outcome of our study was the consistently lower K_fix_ estimates in our control participants than those observed in (25). The K_fix_ coefficient corresponds to the difference between eye velocity component of pursuit during head-restrained and head-unrestrained versions of the task. The authors in that study assert that because the internal program for pursuit is likely to be the same regardless of head restraint, the eye-in-head velocities in these two conditions should be driven by the same visually driven and extraretinal components and should thus be equivalent. The authors do allow, however, “that although [these eye-in-head velocities] share similar dynamic characteristics they may differ in magnitude” given response variabilities in their own data.

### 4.2 Potential mechanisms of altered eye-head coordination in CFL for pursuit

Taken together with our prior findings that gaze velocities are lower during head-unrestrained pursuit (26), lower eye-in-head velocities in the head-unrestrained trials (K_fix_ < 1), and proportionate compensatory responses to head velocities (K_v_ ~ 1) suggest that a potential mechanism for pursuit deficits in CFL is due to: (1) lower eye movement velocities when there is an anticipation of head movement (head-unrestrained condition), particularly in our control cohort, combined with (2) a lack of VOR cancellation in CFL (and to a possibly lesser degree controls) in smooth pursuit.

In terms of the decrease in eye velocity in the head-unrestrained condition, our data suggest that in our cohort of older adults, there is an overall attenuation in eye-driven pursuit when the head is free to move, regardless of CFL. Our analysis of latency offsets between conditions indicates that it is unlikely to be due to a change in pursuit latency between conditions. On the other hand, this eye velocity difference may be indicative of an overall increased reliance on head movements for gaze shifts in older age (37). This interpretation is consistent with the proportional attenuation we see in eye velocity between conditions where regardless of the eye velocity command in the head-restrained condition, there is a similar decrement in velocity across participants and directions. It is possible that this decrement could be due to an additional head pursuit command. Taking this conjecture one step further, a less pronounced change in eye velocities between conditions in our CFL group could suggest that head movements may not be an effective gaze-shifting strategy due to a lack of VOR cancellation in CFL. Thus, the emphasis on head movements is decreased and changes in eye velocity are less systematic between conditions.

Shifting to VOR cancellation, one may argue that the reason we found near-one K_v_ estimates in the CFL group is not a lack of VOR cancellation, but that these participants did not experience target extinction due to its disappearance in the scotoma throughout the trial. If that were indeed the case, their task would be more like the controls’ task in our study or the control task in (24, 25). Indeed, participant P1 (Supplementary Table S&V1 and Table 1 in (26)) had the largest scotoma, lowest pursuit gains, and one of the highest K_v_ estimates (K_v_ = −0.80). This explanation is rather unlikely, however. First, our participants in the head-unrestrained condition had pursuit gains that suggested significant retinal slip (26). Indeed, the participant for whom we observed the greatest amount of VOR cancellation [P5 in (26), K_v_ = −0.66] had one of the smallest binocular scotomas. Second, in our prior work using the scanning laser ophthalmoscope, we documented target disappearance in the scotoma during pursuit as rather common (17). Finally, we have shown previously that saccades during pursuit do not sufficiently make up for shortfalls in displacement (26), likely due to their misdirected nature (20) and were therefore unlikely to effectively keep the target out of the scotoma throughout all trials.

Perceptual filling in in the scotoma (18) may be another consideration. Unlike in the case of target disappearance or an occluder, individuals with CFL are often not aware of their scotoma and instead of a blanking, they experience a filling in of the missing information from their periphery. In this case, they may not be consistently aware that the target disappears during the trial. Indeed, optokinetic nystagmus (OKN) has been shown to be generated by a filling in of an OKN stimulus (38). For pursuit stimuli, however, prior work has shown that individuals with CFL have poorer motion extrapolation across the scotoma (19) and filling in is known to be less effective for non-uniform stimuli (18). Regardless of the specific perception of the target, pursuit gains were significantly below one in our population (26), which would suggest the need for head contribution for improved pursuit.

In their study, Ackerley and Barnes instructed participants to “move the eyes and head” to track the target, whereas we asked the participants to pursue targets in the most natural and comfortable manner possible. As a result, we saw lower head velocities and a greater heterogeneity of pursuit strategies in CFL and control participants. Indeed, our CFL (but not control participants) had a much broader K_v_ range indicating a variable ability to suppress VOR during smooth pursuit. Further, this outcome is not surprising given the variability we see in smooth pursuit gains in CFL during head-restrained pursuit (17, 39) and the similarity in neural control of smooth pursuit and VOR cancellation (40).

Finally, an alternative explanation for the variability in eye-head coordination we saw in our CFL participants is an altered VOR. However, we did not find VOR deficits in a separate cohort of participants with maculopathy (Figure 5). These findings are consistent with prior literature (16) for VOR responses to self-generated head movements for near viewing (0.57 m) and suggest similarly intact vestibular function between the two groups. Thus, any differences we observe in the K_v_ estimates are unlikely due to functional differences in VOR between groups.

### 4.3 Limitations and future work

It is not clear if the lack of VOR cancellation we observe is due to central visual field loss specifically or aging more generally. Prior work has shown that smooth pursuit under head-restrained (41) and unrestrained conditions, as well as the efficacy of VOR cancellation (42, 43) are all impaired in older adults. While we are not provided with the age range of the participants in the Ackerley and Barnes work, it is likely safe to assume that their median age is significantly lower than that in our sample. While we assumed that our controls would behave similarly to the participants in (24, 25) during control trials, having near perfect eye velocities and cancellation of head movements by the VOR, it would be interesting to assess this older cohort’s behavior in trials where the target is temporarily extinguished, and head movements may provide an enhancement of the pursuit response. Future work is needed to help differentiate between the effects of aging generally and age-related changes in visual function.

While the current study sheds light on eye-head coordination during smooth pursuit in CFL, we do not examine VOR cancellation (or adaptation) by itself in this population. While it is well established that individuals with CFL have pursuit deficits, there is very little research available on their ability to adapt their VOR. Demer et al. examined VOR plasticity in response to telescopic magnification in low vision participants and found that a 15-min exposure to magnification was sufficient for low vision participants to exhibit plasticity. However, the study included participants with a variety of vision conditions, and it is difficult to ascertain how those with CFL compared to the rest of the rather broad sample. We do know, however, that more than 50% of those with macular degeneration were not successful in adapting to the use of telescopic lenses (though not necessarily due to insufficient adaptation) (44). Additional work in CFL looking at VOR adaptation without gaze-shifting could help shed light on whether patients are able to sufficiently adapt their VOR gain to the target motion and under what conditions.

Another consideration is the variability in eye-head coordination strategies employed by our participants. We instructed our participants to pursue in a manner that was most natural to them to allow for each participant’s individual adaptation and to understand if some were more effective than others (reliance on head movements versus not). As expected, participants exhibited a wide range of eye-head movement strategies, with some using head movements on almost all the trials, and others relying much more on the eyes alone. Future studies could examine VOR cancellation during pursuit under several different instruction conditions that would compare participants’ natural behaviors versus specific pursuit conditions. Using a larger participant sample and under a range of pursuit instructions would also allow for the analysis of head-velocity matched subsets of participants (or CFL-control matched pairs) to examine whether eye-head coordination strategy might be related to VOR cancellation in either group.

Additionally, the limited sample size and large variability in K_v_ in the CFL group (Figure 3D) could put in question the conclusion that on average CFL participants did not cancel their VOR (K_v_ = −1). While it is similar to that in (24, 25) – seven in the current study versus 6 in the prior publications, and our initial sample size calculation based on prior work (25) suggested that seven participants was sufficient, the variability in our sample exceeded that reported previously (Table 2). Our CFL participants are more heterogeneous in terms of age, visual function, disease progression and adaptation to it. However, our findings are illuminating even with the limited statistical power. Patients with CFL are highly heterogeneous in their oculomotor strategies, and our results indicate that at least a subset of them do not sufficiently cancel their VOR. This finding on its own could point to important future directions for development of training and rehabilitation strategies that would target those individuals who are not able to appropriately leverage head movement to enhance smooth pursuit, or gaze shifting more broadly. Future work that would examine differences between individuals with CFL who do and do not cancel their VOR will be important to understand the source of eye-head coordination deficits and potential ways to address them in this subgroup.

### 4.4 Clinical implications and conclusions

VOR cancellation during smooth pursuit is essential in natural tasks. Understanding why adaptations are evident in some, but not all CFL participants will allow for a development of training strategies that may improve eye-head coordination in this population, allowing for head-based adaptations to address known deficits in target tracking. The lack of VOR cancellation in CFL suggest that these patients are not adapting to their changes in vision, but instead still use the same strategy as their age-matched healthy sighted peers who do not experience target disappearance during the trial. There could be several reasons for this behavior, including (1) a lack of awareness of the target disappearance in their scotoma (45) or an (2) inability to cancel their VOR due to a lack of oculomotor control or VOR cancellation with a non-foveal PRL. For the first, scotoma awareness training has been demonstrated to be effective in individuals with CFL (46) and those with simulated scotomas (47) for other oculomotor tasks.

In terms of the second possibility, the vestibular system’s plasticity and adaptability are some of its key hallmarks, with VOR adaptation being a prime example where VOR gain must change quickly in response to factors such as viewing distance (48) or optical correction (44). While the close links between smooth pursuit and VOR cancellation (28) may provide a pitfall in the trainability of this behavior in CFL, they may also suggest a need for a unified training paradigm for both behaviors. Research in cerebellar disease suggests that VOR cancellation and smooth pursuit may not be as tightly linked as previously thought (49). The complexity of these oculomotor links and heterogeneity of eye-head coordination deficits in CFL suggest the need for developing flexible rehabilitation strategies that would train those with CFL to use head movements more or less depending on how well they can coordinate eye and head movements (determined on individual basis). A consideration of age as a factor will also be important in training approach development.

## Data Availability

The raw data supporting the conclusions of this article will be made available by the authors, without undue reservation.

## References

[1] Klein R Chou C-F Klein BEK Zhang X Meuer SM Saaddine JB. Prevalence of age-related macular degeneration in the US population. Arch Ophthalmol. (2011) 129:75–80. doi: 10.1001/archophthalmol.2010.318, 21220632

[2] Friedman DS O’Colmain BJ Muñoz B Tomany SC McCarty C Jong PTV . Prevalence of age-related macular degeneration in the United States. Arch Ophthalmol. (2004) 122:564–72. doi: 10.1001/archopht.122.4.564 15078675

[3] Mitchell J Bradley C. Quality of life in age-related macular degeneration: a review of the literature. Health Qual Life Outcomes. (2006) 4:97. doi: 10.1186/1477-7525-4-97, 17184527 PMC1780057

[4] Fletcher DC Schuchard RA. Visual function in patients with choroidal neovascularization resulting from age-related macular degeneration: the importance of looking beyond visual acuity. Optom Vis Sci. (2006) 83:178–89. doi: 10.1097/01.opx.0000204510.08026.7f, 16534460

[5] Szabo SM Janssen PA Khan K Lord SR Potter MJ. Neovascular AMD: an overlooked risk factor for injurious falls. Osteoporos Int. (2010) 21:855–62. doi: 10.1007/s00198-009-1025-8, 19629614

[6] Szabo SM Janssen PA Khan K Potter MJ Lord SR. Older women with age-related macular degeneration have a greater risk of falls: a physiological profile assessment study. J Am Geriatr Soc. (2008) 56:800–7. doi: 10.1111/j.1532-5415.2008.01666.x, 18363677

[7] Wood JM Lacherez P Black AA Cole MH Boon MY Kerr GK. Risk of falls, injurious falls, and other injuries resulting from visual impairment among older adults with age-related macular degeneration. Invest Ophthalmol Vis Sci. (2011) 52:5088–92. doi: 10.1167/iovs.10-6644, 21474773

[8] Fuller GF. Falls in the elderly. Am Fam Physician. (2000) 61:2159–68. 10779256

[9] Jager RD Mieler WF Miller JW. Age-related macular degeneration. N Engl J Med. (2008) 358:2606–17. doi: 10.1056/nejmra0801537, 18550876

[10] Schneck ME Haegerstöm-Portnoy G Lott LA Brabyn JA. Monocular vs. binocular measurement of spatial vision in elders. Optom Vis Sci. (2010) 87:526–31. doi: 10.1097/opx.0b013e3181e61a88, 20526225 PMC2928053

[11] Seiple W Szlyk JP McMahon T Pulido J Fishman GA. Eye-movement training for reading in patients with age-related macular degeneration. Invest Ophthalmol Vis Sci. (2005) 46:2886–96. doi: 10.1167/iovs.04-1296, 16043863

[12] Verghese P Vullings C Shanidze N. Eye movements in macular degeneration. Annu Rev Vis Sc. (2021) 7:773–91. doi: 10.1146/annurev-vision-100119-125555, 34038144 PMC8916065

[13] Renninger L Dang L Verghese P Fletcher D. Effect of central scotoma on eye movement behavior. J Vis. (2010) 8:641–1. doi: 10.1167/8.6.641

[14] White JM Bedell HE. The oculomotor reference in humans with bilateral macular disease. Invest Ophthalmol Vis Sci. (1990) 31:1149–61. 2354915

[15] Pidcoe PE Wetzel PA. Oculomotor tracking strategy in normal subjects with and without simulated scotoma. Invest Ophthalmol Vis Sci. (2006) 47:169–78. doi: 10.1167/iovs.04-0564, 16384959

[16] González E Shi R Tarita-Nistor L Mandelcorn E Mandelcorn M Steinbach M. Image stabilization in central vision loss: the horizontal Vestibulo-ocular reflex. Vision. (2018) 2:19–5. doi: 10.3390/vision2020019, 31735883 PMC6835367

[17] Shanidze N Fusco G Potapchuk E Heinen S Verghese P. Smooth pursuit eye movements in patients with macular degeneration. J Vis. (2016) 16:1–1. doi: 10.1167/16.3.1, 26830707 PMC4748745

[18] Zur D Ullman S. Filling-in of retinal scotomas. Vis Res. (2003) 43:971–82. doi: 10.1016/s0042-6989(03)00038-5, 12676241

[19] Rubinstein JF Alcalde NG Chopin A Verghese P. Oculomotor challenges in macular degeneration impact motion extrapolation. J Vis. (2025) 25:17. doi: 10.1167/jov.25.1.17, 39878697 PMC11781323

[20] Shanidze NM Lively Z Lee R Verghese P. Saccadic contributions to smooth pursuit in macular degeneration. Vis Res. (2022) 200:108102. doi: 10.1016/j.visres.2022.108102, 35870286 PMC9831682

[21] Shanidze NM Verghese P. Smooth pursuit deficits impact dynamic visual acuity in macular degeneration. Optom Vis Sci. (2024) 101:435–42. doi: 10.1097/opx.0000000000002144, 38913934 PMC11239305

[22] Bennett SJ Barnes GR. Human ocular pursuit during the transient disappearance of a visual target. J Neurophysiol. (2003) 90:2504–20. doi: 10.1152/jn.01145.2002, 14534275

[23] Barnes GR Collins CJS. The influence of briefly presented randomized target motion on the extraretinal component of ocular pursuit. J Neurophysiol. (2008) 99:831–42. doi: 10.1152/jn.01033.2007, 18057108

[24] Ackerley R Barnes GR. The interaction of visual, vestibular and extra-retinal mechanisms in the control of head and gaze during head-free pursuit. J Physiol. (2011) 589:1627–42. doi: 10.1113/jphysiol.2010.199471, 21300755 PMC3099020

[25] Ackerley R Barnes GR. Extraction of visual motion information for the control of eye and head movement during head-free pursuit. Exp Brain Res. (2011) 210:569–82. doi: 10.1007/s00221-011-2566-6, 21298423 PMC3140921

[26] Shanidze N Velisar A. Eye, head, and gaze contributions to smooth pursuit in macular degeneration. J Neurophysiol. (2020) 124:134–44. doi: 10.1152/jn.00001.2020, 32519572 PMC7474451

[27] Johnston JL Sharpe JA. The initial vestibulo-ocular reflex and its visual enhancement and cancellation in humans. Exp Brain Res. (1994) 99:302–8. doi: 10.1007/bf00239596, 7925810

[28] Barnes GR. Visual-vestibular interaction in the control of head and eye movement: the role of visual feedback and predictive mechanisms. Prog Neurobiol. (1993) 41:435–72. doi: 10.1016/0301-0082(93)90026-O, 8210413

[29] Barnes GR Grealy MA. Predictive mechanisms of head-eye coordination and vestibulo-ocular reflex suppression in humans. J Vestib Res. (1992) 2:193–212. doi: 10.3233/VES-1992-2302, 1342395

[30] Lefevre P Bottemanne I Roucoux A. Experimental study and modeling of vestibulo-ocular reflex modulation during large shifts of gaze in humans. Exp Brain Res. (1992) 91:496–508. doi: 10.1007/bf00227846, 1483522

[31] Rabbitt RD. Semicircular canal biomechanics in health and disease. J Neurophysiol. (2019) 121:732–55. doi: 10.1152/jn.00708.2018, 30565972 PMC6520623

[32] Shanidze N Kim AH Loewenstein S Raphael Y King WM. Eye-head coordination in the guinea pig II. Responses to self-generated (voluntary) head movements. Experimental brain research. (2010) 205:445–454. doi: 10.1007/s00221-010-2375-3, 20697698 PMC2937359

[33] King WM Shanidze N. Anticipatory eye movements stabilize gaze during self-generated head movements. Annals of the New York Academy of Sciences. (2011) 1233:219–225. doi: 10.1111/j.1749-6632.2011.06165.x, 21950997 PMC4049289

[34] Haggerty SE King WM. The Interaction of Pre-programmed Eye Movements With the Vestibulo-Ocular Reflex. Front Syst Neurosci. (2018) 12:4. doi: 10.3389/fnsys.2018.00004, 29593506 PMC5855878

[35] Shanidze N Verghese P. Motion perception in central field loss. J Vis. (2019) 19:20. doi: 10.1167/19.14.20, 31868895 PMC6927672

[36] Guénot J Trotter Y Fricker P Cherubini M Soler V Cottereau BR. Optic flow processing in patients with macular degeneration. Invest Ophthalmol Vis Sci. (2022) 63:21. doi: 10.1167/iovs.63.12.21, 36378131 PMC9672899

[37] Proudlock FA Shekhar H Gottlob I. Age-related changes in head and eye coordination. Neurobiol Aging. (2004) 25:1377–85. doi: 10.1016/j.neurobiolaging.2004.02.024, 15465636

[38] Valmaggia C Gottlob I. Optokinetic nystagmus elicited by filling-in in adults with central scotoma. Invest Ophthalmol Vis Sci. (2002) 43:1804–8. 12036982

[39] Shanidze N Heinen S Verghese P. Monocular and binocular smooth pursuit in central field loss. Vis Res. (2017) 141:181–90. doi: 10.1016/j.visres.2016.12.013, 28057580 PMC5502200

[40] Leigh RJ Zee DS. The neurology of eye movements. 5th ed. Oxford: Oxford University Press (1999).

[41] Sharpe JA Sylvester TO. Effect of aging on horizontal smooth pursuit. Invest Ophthalmol Vis Sci. (1978) 17:465–8. 640792

[42] Kim JS Sharpe JA. The vertical vestibulo-ocular reflex, and its interaction with vision during active head motion: effects of aging. J Vestib Res. (2001) 11:3–12. doi: 10.3233/ves-2001-11102, 11673674

[43] Baloh RW Jacobson KM Socotch TM. The effect of aging on visual-vestibuloocular responses. Exp Brain Res. (1993) 95:509–16. doi: 10.1007/bf00227144, 8224077

[44] Demer JL Porter FI Goldberg J Jenkins HA Schmidt K Ulrich I. Predictors of functional success in telescopic spectacle use by low vision patients. Invest Ophthalmol Vis Sci. (1989) 30:1652–65. 2787302

[45] Fletcher DC Schuchard RA Renninger LW. Patient awareness of binocular central scotoma in age-related macular degeneration. Optom Vis Sci. (2012) 89:1395–8. doi: 10.1097/opx.0b013e318264cc77, 22863789

[46] Janssen CP Verghese P. Training eye movements for visual search in individuals with macular degeneration. J Vis. (2016) 16:29. doi: 10.1167/16.15.29, 28027382 PMC5214619

[47] Biles MK Maniglia M Yadav IS Vice JE Visscher KM. Training with simulated scotoma leads to Behavioral improvements through at least two distinct mechanisms. Invest Ophthalmol Vis Sci. (2023) 64:14. doi: 10.1167/iovs.64.1.14, 36656567 PMC9872837

[48] Snyder LH Lawrence DM King WM. Changes in vestibulo-ocular reflex (VOR) anticipate changes in vergence angle in monkey. Vis Res. (1992) 32:569–75. doi: 10.1016/0042-6989(92)90249-i, 1604844

[49] Takeichi N Fukushima K Sasaki H Yabe I Tashiro K Inuyama Y. Dissociation of smooth pursuit and vestibulo-ocular reflex cancellation in SCA-6. Neurology. (2000) 54:860–6. doi: 10.1212/wnl.54.4.860, 10690977

