# Supplementary material for: Cancellation of the vestibulo-ocular reflex during smooth pursuit in patients with maculopathy
Source: Front Neurol. 2026 Jan 14;16:1632527. doi: 10.3389/fneur.2025.1632527 (PMC12848922; doi:10.3389/fneur.2025.1632527)
Supplement: Supplementary file 1 [file Table_1.DOCX]

**Table S&V1:** Participant summary from Shanidze & Velisar (2020) *J Neurophysiol* (Table 1). PRL for the dominant eye and uncorrected visual acuity are reported.

| **Participant** | **Sex** | **Age** | **Dx** | **Visual Acuity (logMAR)** | **MARS Contrast Sensitivity** | **PRL Distance (°)** |
| --- | --- | --- | --- | --- | --- | --- |
| **P1** | M | 56 | Stargardt's | 1.55 | --- | --- |
| **P2** | F | 79 | AMD (left) | 0 | 0.92 | 0.0 |
| **P3** | F | 77 | AMD | 0.3 | 1.36 | 2.3 |
| **P4** | M | 78 | AMD | 1.1 | 1.36 | 14.7 |
| **P5** | F | 87 | AMD | 0.2 | 1.40 | 6.2 |
| **P6** | F | 77 | AMD | 1.2 | 1.28 | 7.7 |
| **P7** | M | 89 | AMD | 1.2 | 0.88 | 13.0 |
| **P8** | M | 58 | Stargardt's | 0.9 | 1.64 | 5.3 |
| **C1** | M | 61 | --- | 0.9 | 1.8 | 0.0 |
| **C2** | F | 75 | --- | 0.26 | 1.84 | 0.0 |
| **C3** | F | 72 | --- | 0.3 | 1.8 | 0.0 |
| **C4** | F | 77 | --- | 0.1 | 1.76 | 0.0 |
| **C5** | F | 78 | --- | -0.1 | 1.8 | 0.0 |
| **C6** | F | 74 | --- | 0.72 | 1.72 | 0.0 |
| **C7** | F | 70 | --- | 0.22 | 1.8 | 0.0 |

**Table S1**: Additional statistics parameters for analysis of K_fix_ and K_v_ as a function of direction and participant group

|  | **K_fix_** | | | | **K_v_** | | | |
| --- | --- | --- | --- | --- | --- | --- | --- | --- |
|  | **F-stat.** | **p-value** | **Geisser-Greenhouse ε (where applied)** | **η_p_²** | **F-stat.** | **p-value** | **Geisser-Greenhouse ε (where applied)** | **η_p_²** |
| **Direction** | F(1.45, 20.82) = 1.95 | p = 0.17 | ε = 0.48 | η_p_² = 0.08 | F(1.25, 12.89) = 1.96 | p = 0.19 | ε = 0.42 | η_p_² = 0.13 |
| **Participant Group** | F(1, 43) = 0.66 | p = 0.42 | --- | η_p_² = 0.02 | F(1, 12) = 1.10 | p = 0.32 | --- | η_p_² = 0.08 |

**Table S2:** Detailed linear regression information for linear fits of Eye Velocity between the head-restrained and head-unrestrained conditions

|  | **Control** | | | | **CFL** | | | |
| --- | --- | --- | --- | --- | --- | --- | --- | --- |
|  | **Equation** | **95% CI Slope** | **R^2^, p-value** | **F-stat. Slope≠0** | **Equation** | **95% CI of slope** | **R^2^, p-value** | **F-stat. Slope≠0** |
| **0°** | Y = 1.01*X + 1.78 | [0.40, 1.63] | 0.78, 0.008 | F(1,5) = 17.72 | Y = 0.86*X + 2.23 | [0.09, 1.64] | 0.62, 0.04 | F(1,5) = 8.22 |
| **90°** | Y = 1.01*X + 1.88 | [-0.10, 2.12] | 0.52, 0.07 | F(1,5) = 5.50 | Y = 2.23*X - 1.21 | [-4.87, 9.33] | 0.48, 0.31 | F(1,2) = 1.82 |
| **180°** | Y = 1.27*X + 0.083 | [0.65, 1.88] | 0.85, 0.003 | F(1,5) = 27.98 | Y = 1.20*X - 1.22 | [-0.55, 2.94] | 0.38, 0.14 | F(1,5) = 3.10 |
| **270°** | Y = 1.60*X - 2.53 | [0.96, 2.24] | 0.92, 0.002 | F(1,4) = 48.24 | Y = 0.90*X + 1.37 | [0.40, 1.39] | 0.86, 0.007 | F(1,4) = 25.08 |
| **All Directions Combined** | Y = 1.07*X + 1.37 | [0.84, 1.29] | 0.79, <0.0001 | F(1,25) = 95.20 | Y = 0.85*X + 1.67 | [0.42, 1.27] | 0.44, 0.0004 | F(1,22) = 17.17 |
